# Whole Exome Sequencing Identifies a Novel Frameshift Mutation of the WRN Gene in a Werner Syndrome Family and Functional Analysis

**DOI:** 10.1002/mgg3.70118

**Published:** 2025-06-18

**Authors:** Hao Xiong, Haiqing Gao, Jianji Wan, Jieping Xiao, Xiaoqun Luo, Xiuqin Dong, Yueheng Wu, Tao Liu

**Affiliations:** ^1^ Department of Dermatology Guangdong Provincial People's Hospital, Guangdong Academy of Medical Sciences, Southern Medical University Guangzhou China; ^2^ Department of Allergy and Immunology Huashan Hospital, Fudan University Shanghai China; ^3^ Department of Dermatology Huashan Hospital, Fudan University Shanghai China; ^4^ Guangdong Provincial Key Laboratory of South China Structural Heart Disease Guangdong Cardiovascular Institute, Guangdong Provincial People's Hospital, Guangdong Academy of Medical Sciences, Southern Medical University Guangzhou China

**Keywords:** frameshift mutation, Werner syndrome, Werner syndrome RecQ‐like helicase gene, whole exome sequencing

## Abstract

**Introduction:**

Werner syndrome (WS) is a rare recessive disorder characterized by premature aging and metabolic abnormalities. WS is caused by mutations in the WS RecQ‐like helicase gene (WRN), which encodes the WRN RecQ‐like helicase protein. This study aimed to identify the deletion mutation in the WRN gene within the WS family and comprehensively analyze its regulatory role.

**Methods:**

We utilized whole exome sequencing to assess gene mutations in non‐close relatives of two patients with WS. The mutation was further verified using Sanger sequencing. Subsequently, the pathophysiological characteristics of the mutation were examined using Western blotting, subcellular localization determination, conservative analysis, and three‐dimensional (3D) protein structure prediction.

**Results:**

Whole exome sequencing revealed a previously unreported homozygous mutation c.3244delG (p.Val1082Tyrfs*17) within exon 27 of the WRN gene. Sanger sequencing confirmed the presence of a homozygous mutation in the two patients, while a heterozygous mutation was identified in the other six family members. Western blotting revealed that the c.3244delG mutation in the WRN gene resulted in a reduced molecular weight of the mutated WRN protein. Furthermore, subcellular localization experiments revealed that the mutant WRN protein could not be effectively transported to the nucleus. Some studies reported that the mutation exhibits a high conservation rate across various species. The three‐dimensional structure prediction indicates that the mutant WRN protein exhibits a distinct structure compared to the wild‐type protein.

**Conclusions:**

This study identified a frameshift mutation in the WRN gene, which was associated with WS. The subsequent functional analysis revealed the inefficiency of the mutated protein. This study broadens the spectrum of known WRN mutations and enhances the comprehension of WS pathogenesis.

## Graphical abstract

A novel frameshift mutation in the WRN gene resulted in a truncated mutated WRN protein.

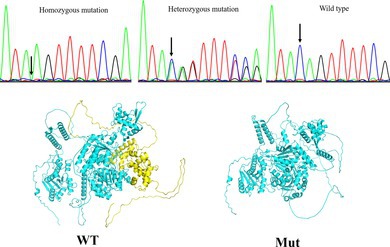

## 1 Introduction

Werner syndrome (WS; OMIM# 277700), first identified by Otto Werner in 1904, is a rare classic segmental progeroid syndrome characterized by sexual traits indicative of accelerated aging (Oshima et al. 2017). WS usually occurs in families with consanguineous relationships, with a global incidence of approximately 1 in 1 million to 1 in 10 million, with a higher incidence of 1 in 100,000 in Japan (Masala et al. 2007). Patients with WS frequently exhibit features of premature aging, including graying hair, alopecia, skin and muscle atrophy, skin changes with ulcers, and typical habits (birdlike face and stunted growth). In addition to natural killer cell receptor deficiency, patients with WS are often accompanied by metabolic abnormalities, including type II diabetes, hypogonadism, osteoporosis, dyslipidemia, and atherosclerosis. Moreover, these patients have an increased risk of cancer, with cancer and myocardial infarction being the predominant causes of death (Lautrup et al. 2019).

WS is caused by the loss of the function of the homozygous or complex heterozygous RECQ‐like helicase gene (WRN). The WRN gene belongs to the human RecQ helicase family and is located on chromosome 8p12. The encoded WRN protein possesses 3′ → 5′ exonuclease and helicase activities (Mukherjee et al. 2018). Increasing evidence indicates that WRN is essential in many pathways that maintain genomic stability, including DNA replication, recombination, repair, telomere maintenance, and transcriptional regulation (Kategaya et al. 2019). These pathways prevent premature aging and tumorigenesis. To date, more than 70 different pathogenic variants were identified in patients with WS from diverse ethnic backgrounds (Oshima and Hisama 2014). However, the mechanism of how WRN mutations induce biochemical alterations and the distinctive pathology of the syndrome remain unclear. Elucidating the function of the WRN gene holds promise for the development of novel therapeutic approaches, targeting not only WS, but also a wide range of cancers and age‐related disorders (Oshima et al. 2017; Paccosi et al. 2025; Jaafar et al. 2022).

In this study, we presented a family from South China diagnosed with WS. We identified a new WRN gene mutation through whole exome sequencing and examined the function and potential pathogenesis of this mutation.

## 2 Materials and Methods

### 2.1 Subjects

The Dermatology Department of Guangdong Provincial People's Hospital, Guangdong Academy of Medical Sciences, is recruiting non‐close family members, and 17 living family members have been included to date.

### 2.2 Peripheral Blood Mononuclear Cells (PBMCs) Isolation and Genomic DNA Extraction

We utilized Ficol‐Paque (GE Healthcare‐Science AB, Uppsala, Sweden) density gradient separation to extract PBMCs from 5 mL of fresh blood anticoagulated with EDTAK2. The purified PBMCs were subsequently processed for genomic DNA isolation using the QIAamp DNA Blood Mini Kit (Qiagen, Hilden, Germany), following the manufacturer's instructions.

### 2.3 Whole Exome Sequencing and Data Analysis

As mentioned earlier, whole exome sequencing is necessary to identify the variation (Yang et al. 2019). The procedure is as follows: The AIExomeV1 kit (iGeneTech Biotech, Beijing, China) is used to capture the exome, followed by high‐throughput sequencing using the Illumina HiSeq 4000 platform (Illumina, San Diego, USA). The average sequencing depth of 100× is adequate to accurately detect variants in 97.4% of the target exome. In the Exome Aggregation Consortium (ExAC), 1000 Genomes project (1000G), Nucleotide Polymorphism, and Genome Aggregation Databases, mutations are excluded when the mutated secondary allele frequency is > 0.01. Variants are screened using SIFT, Polyphen, Mutation Taster, and CADD scores.

### 2.4 Sanger Sequencing

The Sanger sequencing method was utilized to verify WS pathogenic variation in the WRN gene. The prevailing technical standard is to perform polymerase chain reaction (PCR) around candidate pathogenetic mutation loci. The PCR products were purified using Qiagen's QIAquick PCR purification kit (Germantown, MD) and sequenced using ABI Dye Terminator Cycle Sequencing on an ABI3730XL DNA analyzer. The 4.5 Sequencer software (GeneCodes, Ann Arbor, MI) was used to examine the sequence trace files.

### 2.5 Plasmid Construction and Cell Transfection

The coding region of wild‐type and mutated WRN was acquired through synthetic methods. The WRN gene coding sequence was cloned into the BamHI and KpnI loci of vector GV657 (GeneChem Shanghai, China). The GV657 vector was pcDNA3.1‐CMV‐MCS‐3flag‐EF1A‐zsGreen‐sv40‐puromycin. COS‐7 cells were cultured in a sterile incubator at 37°C with 5% CO_2_, utilizing medium model 1640 (Invitrogen), supplemented with 10% fetal bovine serum (Gibco/Thermo Fisher Scientific). The culture bag was placed in a six‐well petri dish during the experiment. The recombinant plasmid was successfully transfected into COS‐7 cells utilizing Lipofectamine 3000 (Invitrogen), following the manufacturer's instructions.

### 2.6 Western Blotting

The Bradford (protein quantification kit, Biyuntian, China) was used to extract and quantify total proteins. Discontinuous sodium dodecyl sulfate‐polyacrylamide gel electrophoresis was employed to separate 50 ng of protein per slot. A 10% acrylamide solution was applied to a polyvinylidene fluoride film. Subsequently, 5% skim milk was maintained at room temperature for 1 h and incubated overnight at 4°C with flag‐labeled monoclonal antibodies (1:1000, Beyotime). Subsequently, tris‐buffered saline with 0.1% Tween and horseradish peroxidase‐conjugated secondary antibodies (dilution factor: 1:800; DAKO) was introduced for incubation. The imprinting was developed utilizing an enhanced chemiluminescence system (Tanon China), following the manufacturer's instructions.

### 2.7 Immunofluorescence

After washing the cells thrice with phosphate‐buffered saline (PBS), they were fixed in 4% paraformaldehyde for 30 min. Subsequently, the cells were washed thrice with PBS and incubated with 0.1% Triton X‐100 (Thermo Fisher Scientific) to render them permeable. After blocking the non‐specific response site, flag‐labeled monoclonal antibodies were incubated overnight at 1:1000. On the second day, after washing the cells thrice with PBS, they were gradually incubated with the species‐specific secondary antibody for 2 h. Subsequently, the nuclei were stained with 4′, 6‐diamino‐2‐phenylindole. The reagent was procured from Sigma Corporation in St. Louis, Missouri, USA. Fluorescence microscopy (Grade: Nikon Eclipse Ti‐U, Tokyo, made in Japan) was utilized to examine transfected cells.

### 2.8 Conservation Analysis and 3D Structure Prediction

The ClustalX 1.81, version 2.1 (http://www.clustal.org/clustal2), was used for the WRN protein sequence analysis of humans, rhesus monkeys, dogs, mice, and elephants. The NCBI genome database (https://www.ncbi.nlm.nih.gov/genome) was used to download the WRN genome. Subsequently, AlphaFold software (version 2.3.2) was employed to predict protein structure and assess its accuracy. PyMoL software (version 2.5.0) was used to plot the predicted structure.

## 3 Results

### 3.1 Clinical Characteristics

**FIGURE 1:**
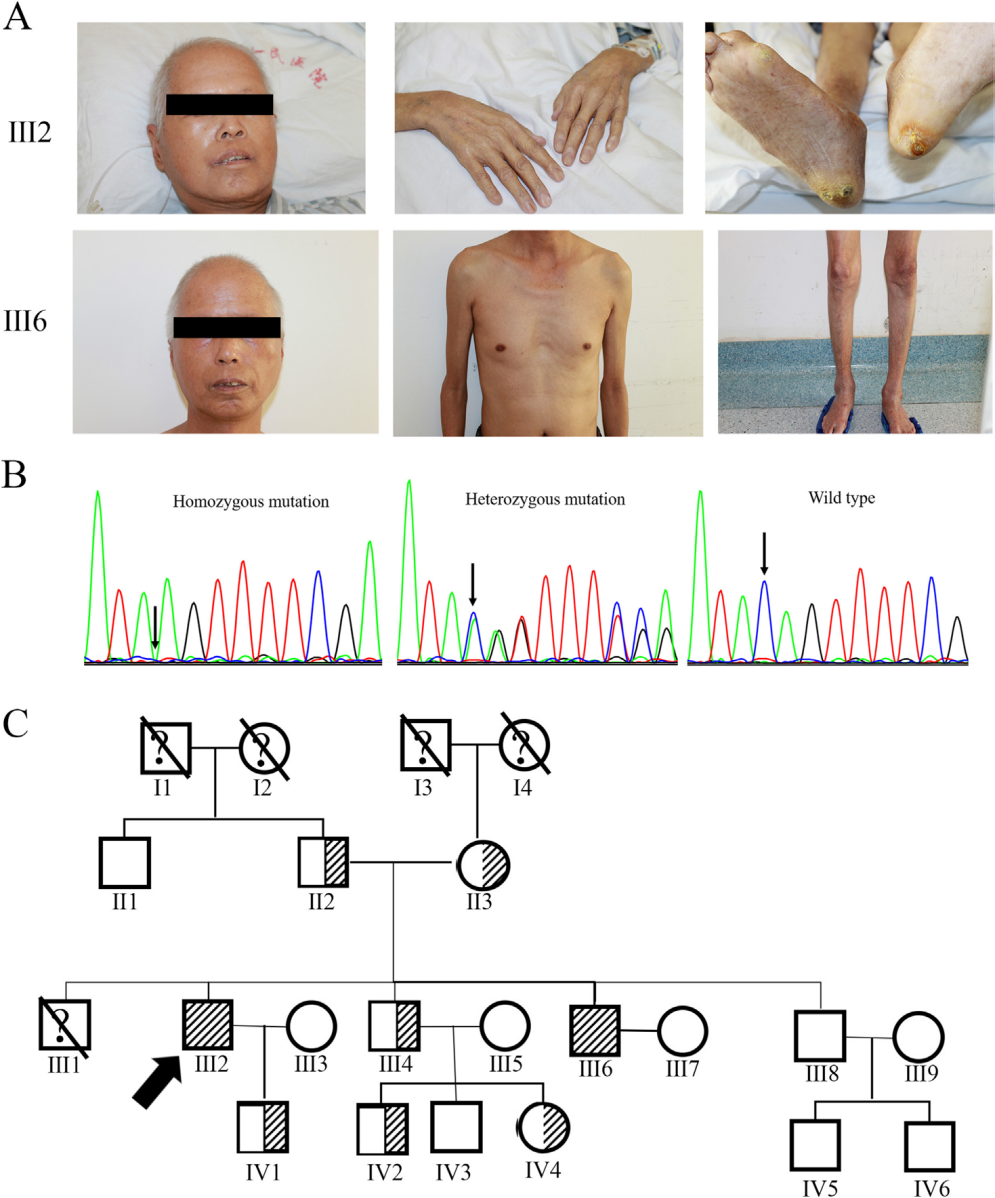
Clinical features of the two patients and mutant sequence of the WRN gene in the family. (A) Photographs of the two patients illustrating the typical WS appearance. (B) Sanger sequencing illustrating a c.3244delG homozygous mutation in the patients and a heterozygous mutation in the family members. (C) Pedigree structure of the family.

The proband (III2), a 54‐year‐old male, presented to our hospital with sclerotic skin. Physical examination revealed that he was malnourished with a hard, birdlike face consistent with scleroderma, a hoarse voice, and ulcers ankles (Figure 1A). One of his younger brothers (III6) exhibited the same appearance (Figure 1A). According to the previously outlined diagnostic criter (Takemoto et al. 2013), these two patients were diagnosed with WS. The remaining family members, including his parents (II2 and II3), two younger brothers (III4 and III8), his son (IV1), nephews (IV2, IV3, IV5, and IV6), and niece (IV4), were phenotypically normal. Laboratory tests conducted on the proband and his brother III included six major tests: complete blood counts, thyroid hormone levels, and liver and kidney functions. No abnormality was detected.

### 3.2 Whole Exome Sequencing Revealed a Novel Mutation in the WRN Gene, and Sanger Sequencing Verified the Mutation in the Family

We performed whole exome sequencing on the proband (III2), another affected member (III6), and normal members (II2, II3, and III8) to investigate gene mutations responsible for the disease. Over 20,000 insertions (indels) and 112,000 single nucleotide polymorphisms (SNPs) were identified in each course. After progressive filtering, we identified a new homozygous mutation c.3244delG (p. Val1082Tyrfs*17) located within exon 27 of the WRN gene in the affected family members (III2 and III6). The heterozygous mutation c.3244delG (p. Val1082Tyrfs*17) in the WRN gene was identified in their parents (II2 and II3), whereas no mutation in the WRN gene was found in their brother (III8).

Sanger sequencing was utilized to confirm the mutation in all the family members. The homozygous mutation c.3244delG (p.Val1082Tyrfs*17) was identified in members III2 and III6, while the heterozygous mutation c.3244delG (p. Val1082Tyrfs*17) was identified in members II2, II3, III4, IV1, IV2, and IV4. In members II1, III3, III5, III7, III8, III9, IV3, IV5, and IV6, the WRN gene exhibited wild‐type status (Figure 1B,C).

### 3.3 Function Analysis of the Mutated WRN Gene

**FIGURE 2:**
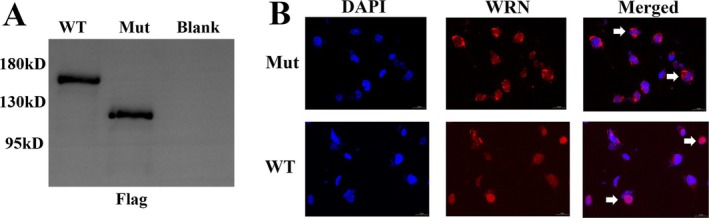
Effect of mutation on WRN function. (A) WRN protein expression in COS‐7 cells transfected with constructs expressing wild‐type and mutated WRN gene using Western blotting. (B) Immunofluorescence of COS‐7 cells transfected with constructs expressing wild‐type and mutated WRN gene for subcellular localization.

We subcloned the mutated WRN gene into a eukaryotic expression vector and transfected it into COS7 cells to comprehensively study the functional effects of WRN gene mutation products. Initially, we performed Western blotting to detect WRN protein expression. We found a reduced molecular weight in the cells transfected with the mutated WRN gene compared to the wild type, indicating that the mutated WRN protein was truncated (Figure 2A, Figure S1). Additionally, we performed immunofluorescence assays to determine the intracellular localization of the gene products. In wild‐type WRN proteins, the protein was completely localized in the nucleus, while in mutant proteins, it was predominantly retained in the cytoplasm (Figure 2B).

### 3.4 Conservation Analysis and 3D Structure Prediction of the Mutated Site

**FIGURE 3:**
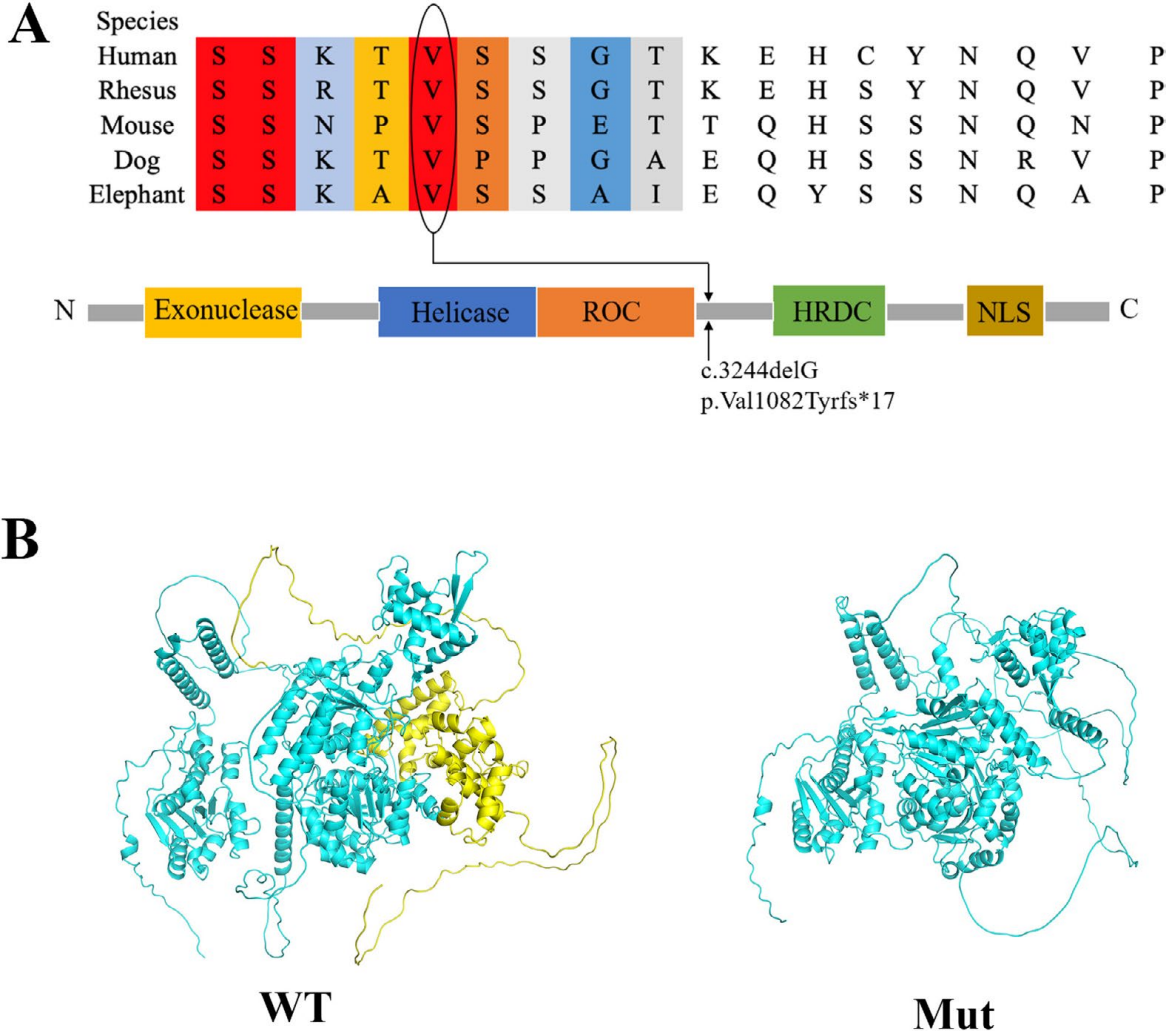
Conservation analysis and structural modeling of this abnormal variation in WRN. (A) The mutation site c.3244delG (p.Val1082Tyrfs*17) was located between the ROC and HRDC regions. The site was highly conserved among different species. RQC, RecQ C‐terminus consensus region; HRDC, RNaseD consensus region; NLS, nuclear localization signal. (B) Structural features of the WRN protein model using AlphaFold (version 2.3.2). The mutated WRN protein lacked the yellow part of the wild‐type protein and demonstrated a different structure.

After examining structural mutation sites in WRN proteins, we evaluated the evolutionary conservation of adjacent sequences and the interspecies crossover. The mutation site is located between the RecQ C‐terminal conserved region of WRN protein and the RNase conserved region. The configuration of the mutated WRN protein was significantly different from that of the wild‐type premature termination protein (Figure 3A), which was confirmed as highly conserved sites across species (Figure 3B).

## 4 Discussion

WS is a rare autosomal recessive, adult‐onset progeroid syndrome. Approximately 1300 patients with WS have been reported globally between the first description by Otto Werner in 1904 and 2002, with over 1000 cases reported in Japan (Masala et al. 2007). A nationwide epidemiological survey in Japan suggests that the high morbidity of WS may be attributed to the relatively high rate of consanguineous marriage within the Japanese population (Takemoto et al. 2013). WS is rarely reported in China. A search on CNKI, WanFang, PubMed, and Web of Science databases revealed that 15 independent WS cases were previously reported in the Chinese population. To our knowledge, this is the first WS family comprising two patients with WS reported in the Chinese population.

The WRN gene belongs to the RecQ team of DNA helicases and comprises 35 exons spanning approximately 250 kb (Yu et al. 1996). Classic WS results from mutations in the WRN gene. Over 80 different disease mutations have been identified in the typical patient with WS, according to the International Werner Syndrome Registry (www.wernersyndrome.org). Mutations encompass base substitutions, insertions, deletions, frameshift mutations, and splicing mutations that interfere with the open reading frame of the WRN. Herein, a family, including two patients suspected of WS, underwent whole exome sequencing, which revealed a homozygous frameshift mutation c.3244delG in exon 27 of the WRN gene in both patients. The outcome was subsequently verified using Sanger sequencing. The parents of the patients were heterozygous carriers of the mutation. This finding facilitated the confirmation of WS diagnosis in this family. The variant has been included in the ClinVar database (Accession: VCV001076156.9) and has been classified as Likely Pathogenic/Pathogenic by multiple submitters. However, related literature reports remain limited. In this study, we identified the variant in a family with clearly defined clinical phenotypes, and the results of co‐segregation analysis further support its potential pathogenicity. This finding provides additional evidence for the clinical significance of this variant within a specific genetic context.

Besides WS, nonsynonymous SNPs in the WRN gene were associated with physiological traits and age‐related diseases, including short stature (Lango Allen et al. 2010), head circumference in infant (Taal et al. 2012), cardiovascular disease (Christoffersen et al. 2017; Luke et al. 2007), dyslipidemia (Kathiresan et al. 2009), diabetes (Hirai et al. 2005), osteoporosis (Mori and Zhou 2016), and ocular cataracts (Jiang et al. 2013). The WRN gene functions as a tumor suppressor gene. The SNPs of the WRN gene are associated with different cancers, including bone and soft tissue sarcomas (Nakayama et al. 2008), meningiomas (Bethke et al. 2008), non‐Hodgkin lymphomas (Guo et al. 2014), lung cancer (Rudd et al. 2006), gastrointestinal neoplasms (Li et al. 2012), breast cancer (Zins et al. 2015), and prostate adenocarcinoma (Wang et al. 2011). The WRN gene exhibits hypermethylation in cancer (Esteller 2007; Agrelo et al. 2006). The modified methylation of the WRN gene may be observed in different tissues according to age (Christensen et al. 2009). Furthermore, the mutation of the WRN gene was also associated with neurodegeneration. Advances in brain imaging have documented brain atrophy in 40% of WS patients, and 10% of WS patients showed clinical signs of schizophrenia (Goto et al. 2013). WRN‐deficient animal models exhibit neurodegenerative features such as cognitive impairment, behavioral deficits, neuroinflammation, and mitochondrial dysfunction, mimicking aspects of aging‐related neural decline (Rekik et al. 2017).

The WRN gene encodes a 180‐kd multifunctional nuclear protein composed of 1432 amino acids (Yu et al. 1996). The WRN protein comprises five functional domains: the exonuclease, helicase, RecQ C‐terminal agreement, RNase agreement, and nuclear localization signal regions (Oshitari et al. 2014). The WRN protein performs 3′ to 5′ helicase activity, ATP‐dependent 3′ to 5′ exonuclease activity, and transactivation functions (Huang et al. 1998). Recently, increasing evidence has indicated that WRN is essential in the pathways that maintain genomic stability and may contribute to protection against cancer and premature aging. Without functional WRN proteins, cells accumulate toxic DNA intermediates or excessively short telomeres, leading to genetic instability, misexpression, and mutagenesis (Dhillon et al. 2007; Eller et al. 2006; Laud et al. 2005). Herein, the frameshift mutation resulted in the substitution of Val with Tyr at the 1082nd amino acid, and the mRNA translation terminated at the 17th amino acid after the mutation site. This mutation resulted in truncation of the WRN protein at the C‐terminus, leading to the loss of the nuclear localization signal region. The nuclear localization signal region is essential for WRN protein targeting the nucleus through the nuclear pore complex (Floch et al. 2015). Consequently, the truncated WRN protein could not translocate into the cell nucleus and was degraded more rapidly within the cell than the wild‐type WRN protein. Based on previous reports, most clinically identified mutations in WRN result in the absence of the nuclear localization signal region at the C‐terminus of the protein. Mutations in the exonuclease domain helicase region have been reported typically in patients with WS who have severe metabolic disorders. In our study, although the patients exhibited typical WS appearance, they exhibited no metabolic abnormalities. This indicated that the exonuclease domain and helicase region are essential for maintaining metabolic function. The mouse models with homozygous deletions in the helicase domain exhibit severe metabolic abnormalities (Labbe et al. 2011). Further studies are required to understand the precise role of residual WRN protein mutations in patients with WS.

The treatment of WS is unclear. Although the clinical management of WS focuses on treatment presentation and complication prevention, new therapeutic approaches have also been investigated in recent years. Hydrogen sulfide can enhance the protein resting state, cellular morphology, and phenotype of WS cells by modulating the mTOR pathway (Talaei et al. 2013). A previous study reported that p38 mitogen‐activated protein kinase inhibitors can reinstate the replication potential of WS fibroblasts (Tivey et al. 2013). Additionally, aminoglycoside and ataluren (ptc‐124) can facilitate full‐length protein expression and restore WRN function in WS cells. However, these medications have not yet been evaluated in patients with WS. The effect of astaxanthin on WS was reported in one case, which significantly improved outcomes for patients with nonalcoholic fatty liver disease due to its anti‐inflammatory and antioxidant capacity (Takemoto et al. 2015).

This study identified a new c.3244delG frameshift mutation in the WRN gene within the WS family. This mutation resulted in the truncation of the WRN protein, consequently impairing its nuclear localization function. The study broadened the spectrum of identified WRN mutations and contributed to the investigation of the molecular biological mechanisms of WS.

## Author Contributions

H.X. and T.L. designed the experiments. H.X., H.G., and J.W. performed the experiments. J.X., X.L., and Y.W. conducted data analysis. H.X. drafted the manuscript. H.X. and X.D. revised the manuscript. T.L. critically reviewed the manuscript.

## Ethics Statement

This study was approved by the Ethics Committee of the Guangdong Provincial People's Hospital (KY‐Z‐2022‐028‐02). The study protocol was conducted in accordance with the Declaration of Helsinki.

## Consent

Informed written consent was obtained from the all‐enrolled family members before the study. All the participants gave written informed consent for their personal or clinical details along with images (including face images) to be published in this study.

## Conflicts of Interest

The authors declare no conflicts of interest.

## Supporting information


Figure S1.

## Data Availability

The datasets generated during the current study are available in the SRA database (PRJNA1174399).
