# Supplementary figures and images for: Whole Exome Sequencing Identifies a Novel Frameshift Mutation of the WRN Gene in a Werner Syndrome Family and Functional Analysis
Source: Mol Genet Genomic Med. 2025 Jun 18;13(6):e70118. doi: 10.1002/mgg3.70118 (PMC12175019; doi:10.1002/mgg3.70118)

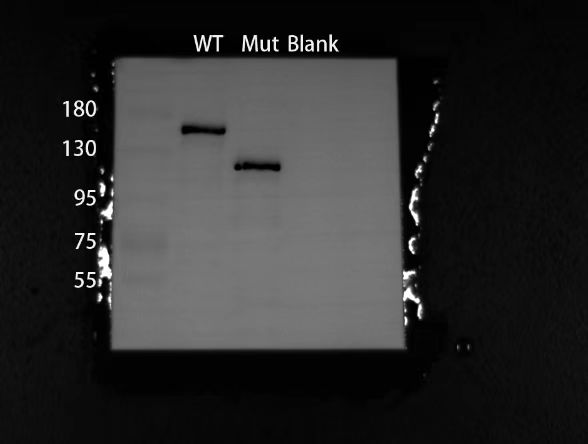

Supplement: Supplementary file 1 — Figure S1. [file MGG3-13-e70118-s001.jpg]
